# Adapting Biased Gene Conversion theory to account for intensive GC-content deterioration in the human genome by novel mutations

**DOI:** 10.1371/journal.pone.0232167

**Published:** 2020-04-30

**Authors:** Rajan Paudel, Larisa Fedorova, Alexei Fedorov

**Affiliations:** 1 Program in Bioinformatics and Proteomics/Genomics, University of Toledo, Toledo, OH, United States of America; 2 CRI Genetics LLC, Santa Monica, CA, United States of America; 3 Department of Medicine, University of Toledo, Toledo, OH, United States of America; University of Leeds, UNITED KINGDOM

## Abstract

We examined seventy million well-characterized human mutations, and their impact on G+C-compositional dynamics, in order to understand the formation and maintenance of major genomic nucleotide sequence patterns. Among novel mutations, those that change a strong (S) base pair G:C/C:G to a weak (W) pair A:T/T:A occur at nearly twice the frequency of the opposite mutations. Such imbalance puts strong downward pressure on overall GC-content. However, along protracted paths to fixation, S→W mutations are much less likely to propagate than W→S mutations. The magnitude of relative propagation disadvantages for S→W mutations is inexplicable by any currently-accepted model. This fact forced us to re-examine the quantitative features of Biased Gene Conversion (BGC) theory. Revised parameters of BGC that, per average individual, convert 7–14 W base pairs into S pairs, would account for the S-content turnover differences between new and old mutations, and make BGC an instrumental force for nucleotide dynamics and evolution. BGC should thus be considered seriously in both theories and biomedical practice. In particular, BGC should be taken into account during allele imputations, where missing SNP alleles are computationally predicted based on the information about several neighboring alleles. Finally, we analyzed the effect of neighboring nucleotide context on the mutation frequencies, dynamics, and GC-composition turnover. For this purpose, we examined genomic regions having extremely biased nucleotide compositions (enriched for S-, W-, purine/pyrimidine strand asymmetry, or AC/GT-strand asymmetry). It was found that point mutations in these regions preferentially degrade the nucleotide inhomogeneities, decreasing the sequence biases. Degradation of sequence bias is highest for novel mutations, and considerably lower for older mutations (those widespread across populations). Besides BGC, there may be additional, still uncharacterized molecular mechanisms that either preserve genomic regions with biased nucleotide compositions from mutational degradation or fail to degrade such inhomogeneities in specific chromosomal regions.

## Introduction

GC-composition along mammalian chromosomes is highly uneven. There are regions highly enriched for G and C nucleotides, and other regions in which these nucleotides are strongly under-represented. This non-randomness has an intricate genomic structure and has been reviewed in many articles [1–3]. Regions with a particular GC-content may expand over millions of base pairs (known as isochores), or occupy only a few hundreds of base pairs and represent small islands inside regions with different GC-composition (some CpG-islands may exemplify the latter cases)[4]. Density, exon-intron structure, and functioning of genes strongly depend on GC-content of chromosomal region they occupy [3, 5]. Moreover, GC-composition is critical for the frequency of DNA recombination [6] and mobility of DNA repeats [7]. Dramatic variations of GC-content are also described in prokaryotes [8, 9] and various eukaryotic species [10, 11].

About 5% of the mammalian genome is represented by various sequences having extreme nucleotide compositions. They include chromosomal segments with extremely AT-rich content or GC-rich content. In addition, they include DNA sequences in which one strand is purine-rich, AC-rich, or highly periodic with alternating purine/pyrimidine sequences. We called these profound biases in nucleotide composition Genomic Mid-Range Inhomogeneity (or Genomic-MRI) [12, 13]. Genomic-MRI regions may form special DNA structures (*e*.*g*., H-DNA, Z-DNA) and they are non-randomly distributed along the genome [14]. At least some of them have well-documented biological roles [13]. Here, we have investigated Genomic MRI regions to quantitate the effect of mutations inside them. Mutations may decrease or increase the nucleotide bias in these regions. For example, A→C, A→G, T→C, and T→G mutations increase GC-composition in GC-rich sequences; G→A, G→T, C→A, and C→T decrease the GC-composition; while A→T, T→A, G→C, and C→G are neutral to GC-richness. We examined how mutations change genomic-MRI regions.

Every human has an average of 50–100 *de novo* mutations, absent in the genomes of the two parents [15]. A majority of *de novo* mutations occur inside non-polymorphic genomic sites in a human population and create brand new very rare polymorphic derived alleles, so-called singletons, which frequency is minimal. A vast majority of such novel mutations will be washed out from the population due to genetic drift. Yet, a minor fraction of them will be propagated from generation to generation until they completely replace ancestral counterpart alleles. This process is stochastic, yet the average time for a neutral mutation from the arrival to the fixation is calculated by a simple formula deduced by Kimura and Ohta [16]: G = 4N_e_, where N_e_ is the effective size of the population (for human European populations N_e_ = 10,000), and G is the average number of generations required for the fixation of novel mutation [17, 18]. Assuming that G for humans is 25 years, then it takes on average about one million years for a neutral mutation to be fixed among humans. Mutations that exist in a population around half a million years or more we call “old”. In this paper we examine the difference in distributions between “old” mutations and “novel” mutations, which arrived a few thousand years ago. To see dynamics of mutation aging we divided derived alleles into five bins based on their frequencies: 0–20%, 20–40%, 40–60%, 60–80%, and 80–100%. According to neutral theory of evolution the propagation of neutral mutations through generations is purely stochastic without any specific acceleration/deceleration segments on the fixation paths [19]. Thus, at the first approximation, it requires about 200,000 years for a mutation to move from one bin into the next one. The number of derived alleles in the first bin (0–20%) is dozens of times more than in the next bins. There is a chance that some old mutations move backward from top bins into the first bin. Yet, such “old” derived alleles, that stochastically appear in the first bin again, are in minority and over-numbered dozens of times by “novel” mutations in the first bin. It gives us a reasonable approximation to consider rare derived alleles in the first bin as “novel” mutations and those in the last two bins as “old” ones.

This influx of novel mutations degrades information that is stored in the DNA sequences and, at the same time, provides an opportunity for creation of useful new genetic information. Currently, over one hundred million human mutations have been characterized in public databases [20–22]. The dynamics of mutations have been investigated for decades, via both experiments and sophisticated mathematical models, yet our understanding of genome evolution is still ambiguous [23]. Each nucleotide type (A, G, C, or T) has specific likelihoods of mutating into another nucleotide type. Mutational preferences also depend on the context created by neighboring nucleotides around the mutation site, and by DNA methylation [24, 25]. Together, these patterns of mutation frequencies create an intricate non-randomness in the genome’s nucleotide composition.

As we showed in this paper, in humans novel mutations that replace G:C base pairs with A:T pairs are a third more frequent than the opposite mutations. This effect appears more prominent if we take into account that the total number of G:C base pairs is 1.38 times lower than the number of A:T pairs. Such mutational bias should create progressively fewer G:C pairs in genomes, from generation to generation, until they reach equilibrium at 34% GC-composition. However, the GC-percentage of the human genome is 42% and, likely, is close to the point of equilibrium [2]. For over twenty years, there has been a strong belief that Biased Gene Conversion (BGC) is important for shaping GC-content in genomes of mammals and other eukaryotes. The BGC theory is based on DNA repair processes inside heteroduplexes–double-stranded DNA segments formed during meiosis at crossover and non-crossover recombination sites [6, 26–28]. One DNA strand of heteroduplexes has maternal origin, while the complementary strand is paternal. The heterozygous sites in heteroduplexes create mismatches some of which may be A:G, A:C, T:G, or T:C. Mismatch repair machinery may convert these mismatches either to strong (S, G:C) or weak (W, A:T) base pairs. BGC theory claims that there is a bias in mismatch repair toward converting these four kinds of mismatches into strong base pairs. Original estimates of this bias in the mammalian genome were very weak: 50.6% (W→S) *vs*. 49.4% (S→W) [29]. However, in 2015 this bias in the humans was re-evaluated, and dramatically increased to over twofold 68% vs. 32% [30]. Our lab also re-evaluated it to the 56% vs. 44% ratio based on the 1000 Genomes public dataset [31]. However, the known quantitative traits and parameters for BGC are unable to explain phenomenon of preservation of GC-composition from degradation by novel mutations. In this paper we re-evaluated the BGC characteristics and outcome to make it consistent with observed genomic GC-composition.

## Results

### Characterization of novel and “old” mutations

The 1000 Genomes Project provides the information about ancestral/mutant status of alleles for a majority of SNPs [22]. From this database we used only those SNPs for which ancestral/mutant alleles were determined with the highest certainty. All SNPs with validated ancestral/mutant status were divided into five bins based on the mean frequency of mutant alleles across continents (averaged frequency within 26 populations from 1000 Genomes). Bins were the following: 0–20% frequency of mutant alleles; 20–40%; 40–60%; 60–80%; and 80–100%. The numbers of SNPs inside these five bins are highly uneven since the distribution of number of SNPs by their mutant allele frequency in populations is exponential (see Extended Data Fig 3 *Auton et al*. [22]). More than half of all known SNPs have rare mutant alleles, where frequencies are <1%. Therefore, the first bin (0–20%) contains 40 times more SNPs than the next bin and about 100 times more SNPs than the fourth bin (See Table 1). Moreover, the vast majority of SNPs in the first (0–20%) bin have rare mutant alleles. It gives us justification for considering mutant alleles from the first bin as being “novel”, to a good approximation.

**Table 1:** Number of different types of point mutations in the whole human genome.

Ancestral	Derived	Bin 1	Bin 2	Bin 3	Bin 4	Bin 5
**allele**	**allele**	**0–20%**	**20–40%**	**40–60%**	**60–80%**	**80–100%**
A	C	2,405,968	57,140	35,497	26,240	49,029
A	G	9,075,719	213,833	135,079	102,057	362,250
A	T	2,264,273	52,006	31,657	22,897	44,943
C	A	3,128,072	66,896	39,210	27,789	47,531
C	G	2,875,987	63,504	37,618	26,810	45,204
C	T	12,795,576	278,973	163,028	113,000	218,117
G	A	12,858,986	279,579	163,179	113,212	218,908
G	C	2,847,258	63,783	37,994	27,143	47,102
G	T	3,090,046	65,309	38,970	27,348	46,520
T	A	2,273,976	52,234	31,856	22,918	44,971
T	C	9,038,949	214,179	134,081	101,441	357,926
T	G	2,395,689	56,219	34,686	25,722	47,088
W	S	22,916,325	541,371	339,343	255,460	-
S	W	31,872,680	690,757	404,387	281,349	-
*ratio*: *(S->W)/(W->S)*		*1*.*391*	*1*.*276*	*1*.*192*	*1*.*101*	-
*SD for (S->W)/(W->S)*		*0*.*0002*	*0*.*002*	*0*.*003*	*0*.*003*	-

Mutations are distributed among five bins based on the frequency of their mutant (derived) allele.

#### G:C vs A:T mutational dynamics in the entire genome

**Fig 1:**
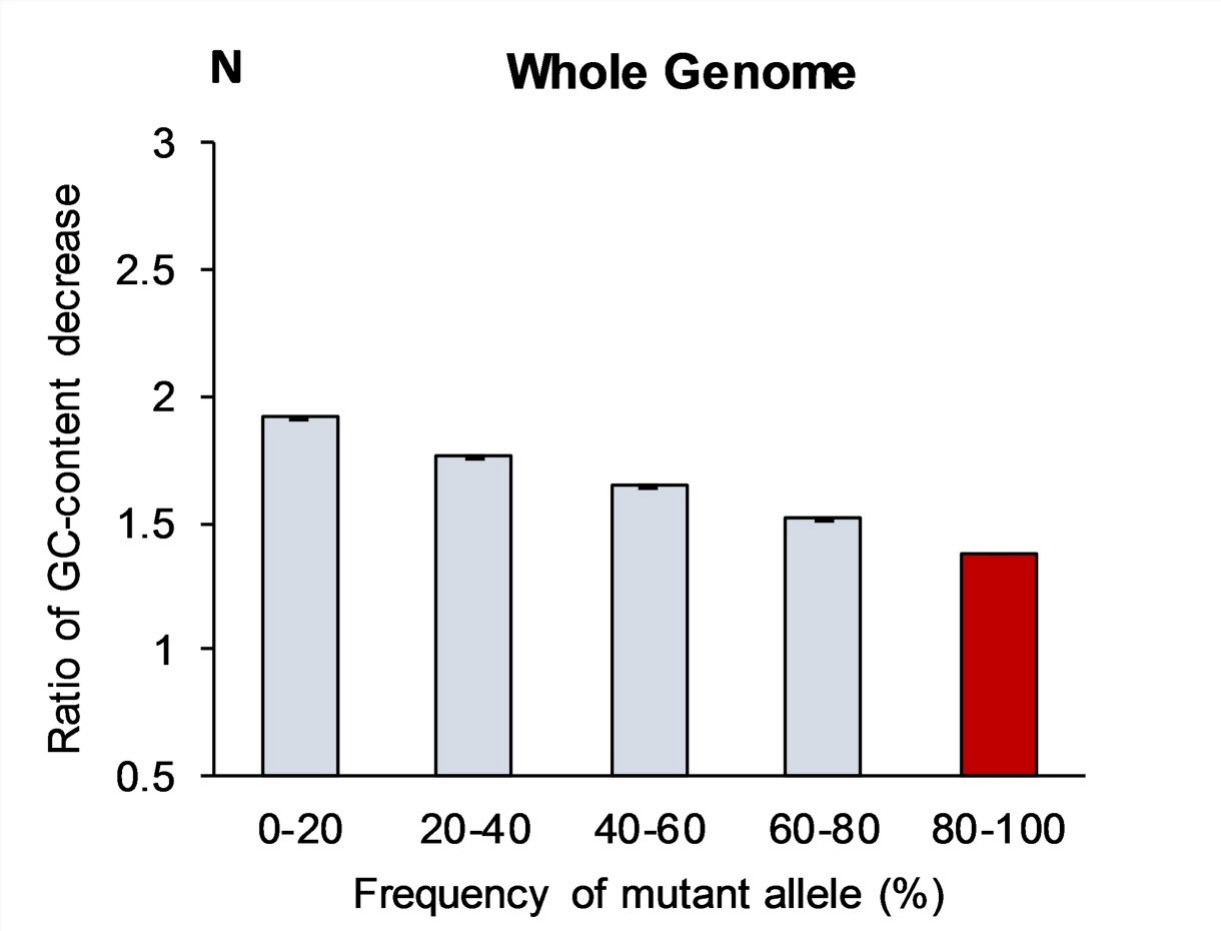
Normalized ratio of GC-content decrease (N) in the whole genome caused by mutations with various frequencies across populations. The N-ratio is the proportion between number of mutations that change S->W and number of W->S mutations normalized for the equal number of S and W sites. The projected N-value for the fifth bin (80–100%) is shown in red, which does not have experimental support. Bars show standard error of the means, which is due to the limited sample size (see Methods section).

Table 1 presents the observed numbers of all types of point mutations, having validated ancestral/mutant status, throughout the entire human genome. The bottom two rows of this table show the aggregate numbers of all kinds of mutations that change strong base pairs into weak ones, and *vice versa*. As explained in the Methods section, misidentification errors for the ancestral/mutant allele status are predominantly concentrated inside 80–100% bin. For this reason, we excluded the data for this last bin from all tables except rows 1–12 in Table 1. Instead, in our figures we projected the observed trends from the first four bins into the fifth one to get an idea what should may be expected inside the last (80–100%) bin. For novel mutations (first bin 0–20%), the prevalence of G:C→A:T over A:T→G:C is the strongest. In this bin the ratio for aggregate number of S→W mutations over W→S is ***R***_(GC→AT)/(AT→GC)_ = 1.39. Novel mutations that convert S→W are more often than W→S mutations despite number of G:C pairs is 1.38 times less abundant than A:T pairs in the human genome. After normalization for equal numbers of G:C and A:T pairs, the exchange S<–>W ratio for novel mutations per base pair becomes ***N***_(GC→AT)/(AT→GC)_ = 1.92. The normalization means that if we consider the same numbers of S and W base pairs, then mutations S→W will be 1.92 times more often than W→S ones in these observed nucleotide sites. This normalization will allow us to compare the results in chromosomal regions with different nucleotide compositions (see next paragraphs). Table 1 and Fig 1 demonstrate that the prevalence of G:C→A:T over A:T→G:C mutations monotonously declines in each consecutive bin, with the increase of mutant allele frequencies, reaching ***R***_(GC→AT)/(AT→GC)_ = 1.10 value for the fourth (60–80%) bin.

If we project this monotonous trend into the last fifth bin (80–100%), then the prevalence of G:C→A:T mutations practically reaches the equilibrium stage ***R***_(GC→AT)/(AT→GC)_ ≅1.0 at which the number of A:T→G:C become practically equal to G:C→A:T for the entire human genome (Fig 1). This implies that the human genome GC-composition at present is frozen, at the current equilibrium of 42% GC.

We also excluded from consideration SNPs inside the most mutable dinucleotides, CpG sites, in order to evaluate their contribution on the observed phenomenon. CpG is the preferred site for mammalian DNA methylation, spontaneous deamination of 5-methylC leads to T, yielding a high rate of CpG→TpG (or CpA) substitutions. We found that C^5me^→T mutations at CpG sites do not noticeably influence on the elevated rate of S→W over W→S for novel mutations and also the equilibrium between A:T→G:C and G:C→AT for “old” mutations (results are in S1 Table).

For better understanding mutational dynamics, we also analyzed mutations inside all DNA repetitive elements that occupy 40% of the human genome. These data are presented in the S2 Table. Inside DNA repeats the selection forces may not exist or at least be considerably smaller than in the coding or regulatory regions. Inside DNA repeats (S2 Table) we observed very similar trend for S <-> W dynamics as in the whole genome (Table 1).

We investigated whether significantly different proportions ***R***_(S→W)/(W→S)_ for novel and “old” mutations may be explained by Biased Gene Conversion (BGC) theory. According to a Duret and Galtier, BGC theory expects drastically different effect of conversions G:C→A:T and A:T→G:C in chromosomal regions with different GC-compositions [32]. The following paragraphs (and Tables 2–5) explore the G:C<–>A:T mutational changes in genomic regions with various biased nucleotide compositions in order to examine whether BGC is responsible for the observed phenomenon.

**Table 2:** Number of different types of point mutations in GC-rich regions (76% GC-content).

Ancestral	Derived	Bin 1	Bin 2	Bin 3	Bin 4
**allele**	**allele**	**0–20%**	**20–40%**	**40–60%**	**60–80%**
A	C	2,405,968	57,140	35,497	26,240
A	G	9,075,719	213,833	135,079	102,057
A	T	2,264,273	52,006	31,657	22,897
C	A	3,128,072	66,896	39,210	27,789
C	G	2,875,987	63,504	37,618	26,810
C	T	12,795,576	278,973	163,028	113,000
G	A	12,858,986	279,579	163,179	113,212
G	C	2,847,258	63,783	37,994	27,143
G	T	3,090,046	65,309	38,970	27,348
T	A	2,273,976	52,234	31,856	22,918
T	C	9,038,949	214,179	134,081	101,441
T	G	2,395,689	56,219	34,686	25,722
W	S	22,916,325	541,371	339,343	255,460
S	W	31,872,680	690,757	404,387	281,349
*ratio*: *(S->W)/(W->S)*		*1*.*391*	*1*.*276*	*1*.*192*	*1*.*101*
*SD for (S->W)/(W->S)*		*0*.*0002*	*0*.*002*	*0*.*003*	*0*.*003*

**Table 3:** Number of different types of point mutations in AT-rich regions (87% AT-content).

Ancestral	Derived	Bin 1	Bin 2	Bin 3	Bin 4
**allele**	**allele**	**0–20%**	**20–40%**	**40–60%**	**60–80%**
A	C	2,453	99	65	37
A	G	8,928	345	214	133
A	T	3,999	178	105	89
C	A	985	50	20	23
C	G	816	23	18	15
C	T	2,920	105	63	50
G	A	3,001	118	70	55
G	C	668	23	17	10
G	T	1,140	36	24	27
T	A	3,946	178	106	76
T	C	9,048	333	220	171
T	G	2,483	112	61	52
W	S	22,912	889	560	393
S	W	8,046	309	177	155
*ratio*: *(S->W)/(W->S)*		*0*.*35*	*0*.*35*	*0*.*32*	*0*.*39*
*SD for (S->W)/(W->S)*		*0*.*005*	*0*.*02*	*0*.*03*	*0*.*04*

**Table 4:** Number of different types of point mutations in R-rich DNA strands (86% R-content).

Ancestral	Derived	Bin 1	Bin 2	Bin 3	Bin 4
**allele**	**allele**	**0–20%**	**20–40%**	**40–60%**	**60–80%**
A	C	6,866	221	120	99
A	G	27,909	1,094	710	482
A	T	4,794	100	79	48
C	A	2,578	91	40	46
C	G	2,175	88	40	33
C	T	9,627	244	165	99
G	A	37,543	1,139	744	495
G	C	10,000	271	180	110
G	T	8,663	197	119	90
T	A	1,675	63	61	27
T	C	6,130	152	111	85
T	G	1,856	56	41	31
W	S	42,761	1,523	982	697
S	W	58,411	1,671	1,068	730
R	Y	30,323	789	498	347
Y	R	8,284	298	182	137
*ratio*: *(S->W)/(W->S)*		*1*.*37*	*1*.*10*	*1*.*09*	*1*.*05*
*ratio*: *(R->Y)/(Y->R)*		*3*.*66*	*2*.*65*	*2*.*74*	*2*.*53*
*SD for (S->W)/(W->S)*		*0*.*01*	*0*.*04*	*0*.*05*	*0*.*06*
*SD for (R->Y)/(Y->R)*		*0*.*04*	*0*.*2*	*0*.*2*	*0*.*3*

**Table 5:** Number of different types of point mutations in AC-rich regions (81% A+C-content).

Ancestral	Derived	Bin 1	Bin 2	Bin 3	Bin 4
**allele**	**allele**	**0–20%**	**20–40%**	**40–60%**	**60–80%**
A	C	3,647	195	114	79
A	G	6,197	200	151	85
A	T	1,594	56	32	23
C	A	3,583	145	119	91
C	G	2,642	88	53	36
C	T	10,976	271	177	132
G	A	4,106	132	68	55
G	C	810	27	34	21
G	T	664	11	10	7
T	A	698	36	28	16
T	C	2,442	130	106	66
T	G	524	21	8	6
W	S	12,810	546	379	236
S	W	19,329	559	374	285
A or C	G or T	21,409	615	413	276
G or T	A or C	8,056	325	236	158
*ratio*: *(S->W)/(W->S)*		*1*.*51*	*1*.*02*	*0*.*99*	*1*.*21*
*ratio*: *(AC->GT)/(GT->AC)*		*2*.*66*	*1*.*89*	*1*.*75*	*1*.*75*
*SD for (S->W)/(W->S)*		*0*.*02*	*0*.*06*	*0*.*07*	*0*.*11*
*SD for (AC->GT)/(GT->AC)*		*0*.*03*	*0*.*1*	*0*.*1*	*0*.*2*

#### Mutational dynamics in GC-rich and AT-rich regions

Table 2 indicates that inside the most GC-rich genomic fragments the frequency of novel mutations (first bin) also favor G:C→A:T over AT→GC changes, with about the same normalized ratio per site (***N***_(GC→AT)/(AT→GC)_ = 2.10) as for the whole genome. [Note that the ***R***_(GC→AT)/(AT→GC)_ = 6.56 is extremely high due to three times overabundance of G+C over A+T nucleotides.]

**Fig 2:**
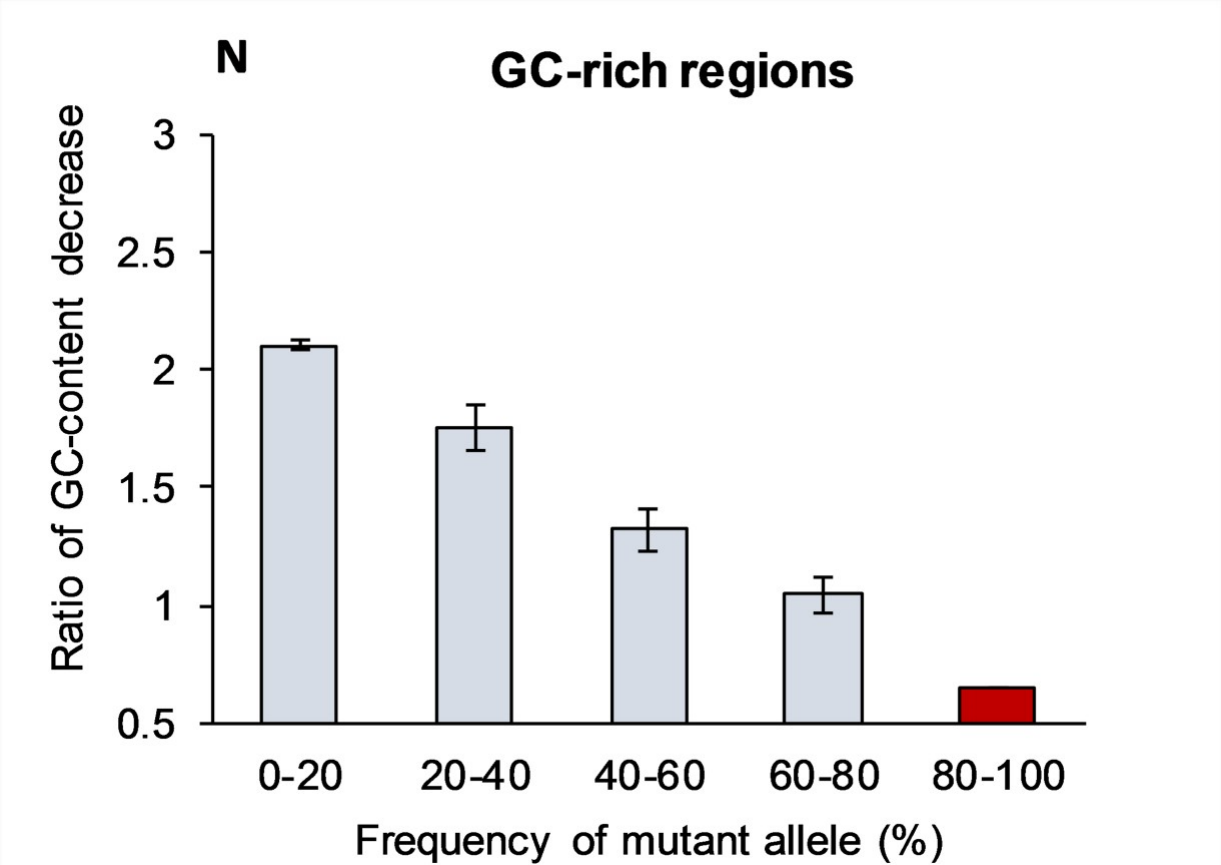
Normalized ratio of GC-content decrease (N) in the GC-rich regions caused by mutations with various frequencies across populations. The N-ratio is the proportion between number of mutations that change S->W and number of W->S mutations normalized for the equal number of S and W sites. The projected N-value for the fifth bin (80–100%) is shown in red, which does not have experimental support. Bars show standard error of the means, which is due to the limited sample size (see Methods section).

This ratio also rapidly and monotonously decreases for the “older” mutant alleles (Fig 2). In the fourth bin with “old” mutations (60–80% of mutant allele frequencies) the normalized ratio per site becomes ***N***_(GC→AT)/(AT→GC)_ = 1.04 (the ***R***_(GC→AT)/(AT→GC)_ = 3.26 for this bin), which is in a good agreement with BGC theory. Fig 2 demonstrates that the projected ratio for the nearly-fixed mutations (80–100%) becomes less than one: ***N***_(GC→AT)/(AT→GC)_ ≅0.7 (the ***R***_(GC→AT)/(AT→GC)_ ≅2.1).

However, since we are considering extremely GC-rich sequences, this ratio for near-fixation stage (***N*** = 0.7) is not enough to prevent 75% GC-richness from mutational degradation (observe that ***R*** is still 2.1); it only slows down the CG-degradation about three-fold.

**Fig 3:**
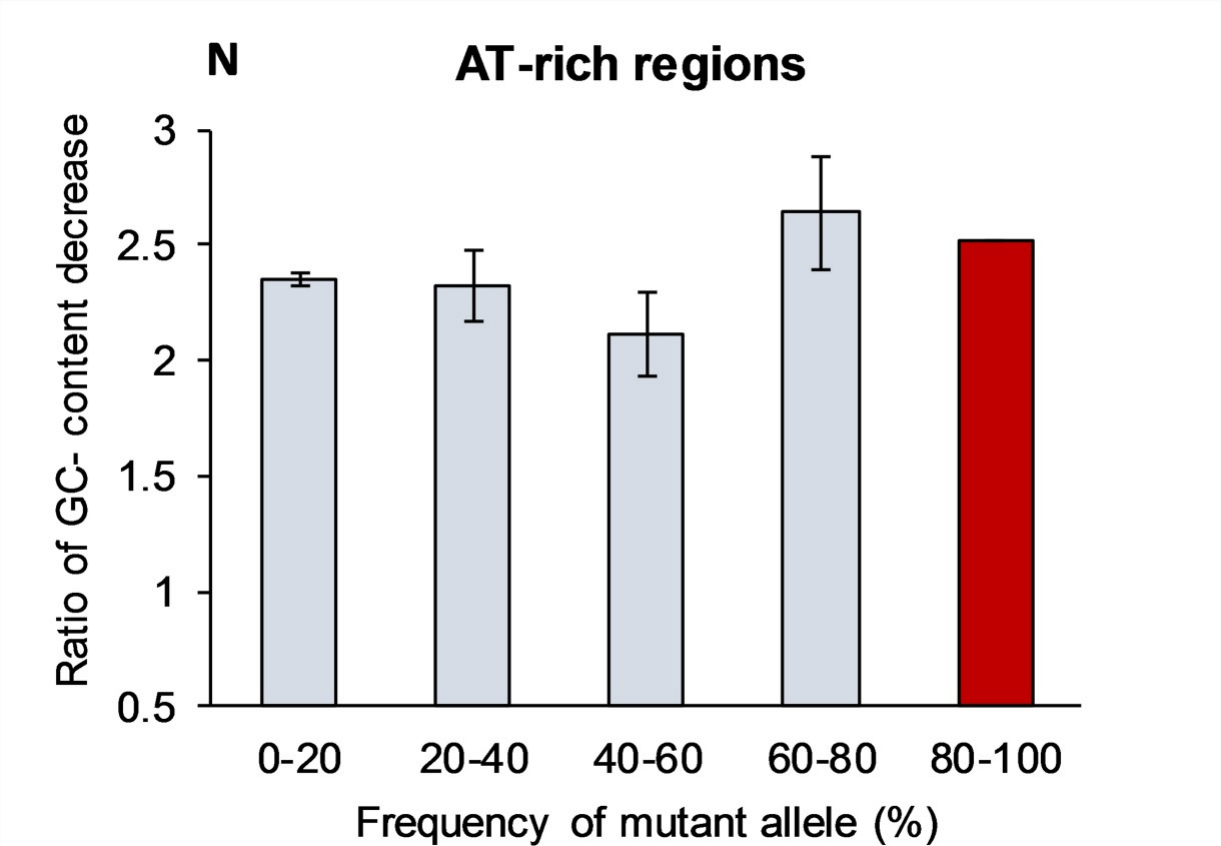
Normalized ratio of GC-content decrease (N) in the AT-rich regions caused by mutations with various frequencies across populations. The N-ratio is the proportion between number of mutations that change S->W and number of W->S mutations normalized for the equal number of S and W sites. The projected N-value for the fifth bin (80–100%) is shown in red, which does not have experimental support. Bars show standard error of the means, which is due to the limited sample size (see Methods section).

Table 3 shows that novel mutations within AT-rich regions also strongly favor converting G:C into A:T pairs. For the first bin, the normalized mutation rate per site in AT-rich regions is ***N***_(GC→AT)/(AT→GC)_ = 2.35 (the ***R***_(GC→AT)/(AT→GC)_ = 0.35 due to seven-fold overabundance of A+T nucleotides over G+C). However, this ratio stays about the same for all bins with different mutant allele frequencies (see Fig 3). For example, for the fourth bin (60–80%) the ***N***_(GC→AT)/(AT→GC)_ = 2.64 and ***R***_(GC→AT)/(AT→GC)_ = 0.39. Such essentially unchanged ***N***-ratio may not contradict BGC, if there are practically no meiotic recombinations inside AT-rich regions including non-crossover cases. The data in Table 3 for AT-rich regions shows that mutations constantly degrade AT-richness by increasing their GC-content. In this process there is no difference between novel and “old” mutations.

#### Mutational dynamics in purine-rich regions

In humans and other species there are distinct genomic regions in which one of the DNA strands is highly purine (R)-rich, while the complementary strand is of course pyrimidine (Y)-rich. We examined mutational dynamics in purine-rich DNA strands, half of which are on the reference DNA (plus) strand, while another half are on the complementary (minus) strand of the human genome (Table 4). In addition to S<->W dynamics, we analyzed frequencies of mutations that change R to Y and vice versa in these R-rich DNA strands. Novel mutations in R-rich regions (Table 4) are in favor of converting S into W base pairs ***R***_(GC→AT)/(AT→GC)_ = 1.37 (since GC% in these regions is 50%, then ***R*** *=* ***N***). For “older” mutations (Bins 2–4) the ***R***-ratio is 30% less, which is statistically significant (see Table 4). This table illustrates that nucleotide neighboring content may influence on the dynamics of mutation appearance and propagation. For example, A->C type of mutation is about 40% more frequent than A->T type in Table 4.

**Fig 4:**
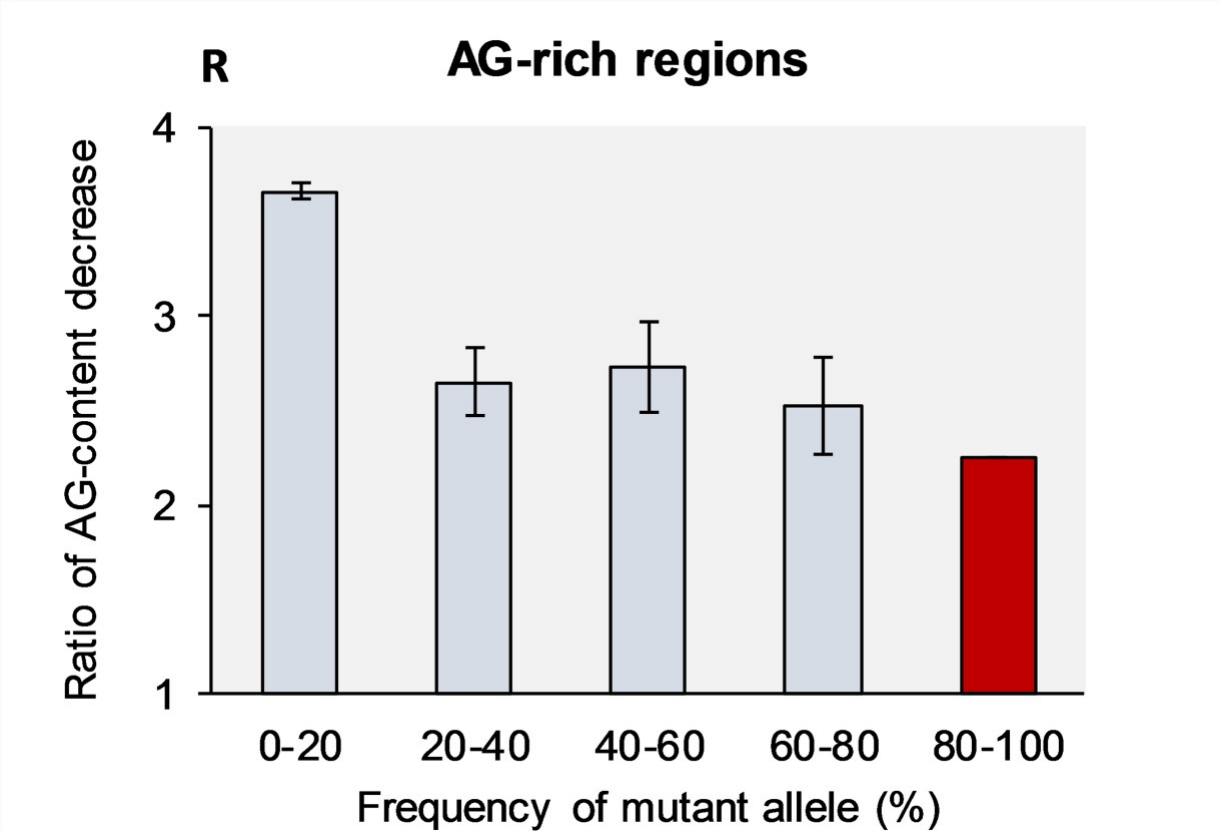
Ratio of purine-content decrease (R) in the purine-rich regions caused by mutations with various frequencies across populations. The R-ratio is the proportion between total number of mutations that change R->Y vs. Y->R mutations. The projected **R**-value for the fifth bin (80–100%) is shown in red, which does not have experimental support. Bars show standard error of the means, which is due to the limited sample size (see Methods section).

Table 4 and Fig 4 show that novel mutations (first bin) most strongly degrade the non-randomness of nucleotide composition of R-rich regions, driving down their purine levels toward average expectancies (the ratio of purine degradation is ***R***_(R→Y)/(Y→R)_ = 3.7 in the first bin).

The “older” mutations have noticeably less effect on degradation of R-richness (***R***_(R→Y)/(Y→R)_ = 2.5 in the fourth bin). For the mutations at the nearly-fixation stage (projected fifth bin (80–100%)), the degradation of R-richness is about 28% smaller than for novel mutations (Fig 4). BGC theory cannot explain why novel mutations degrade R-richness more actively than “old” mutations inside purine-rich regions.

### Mutational dynamics in AC-rich regions

**Fig 5:**
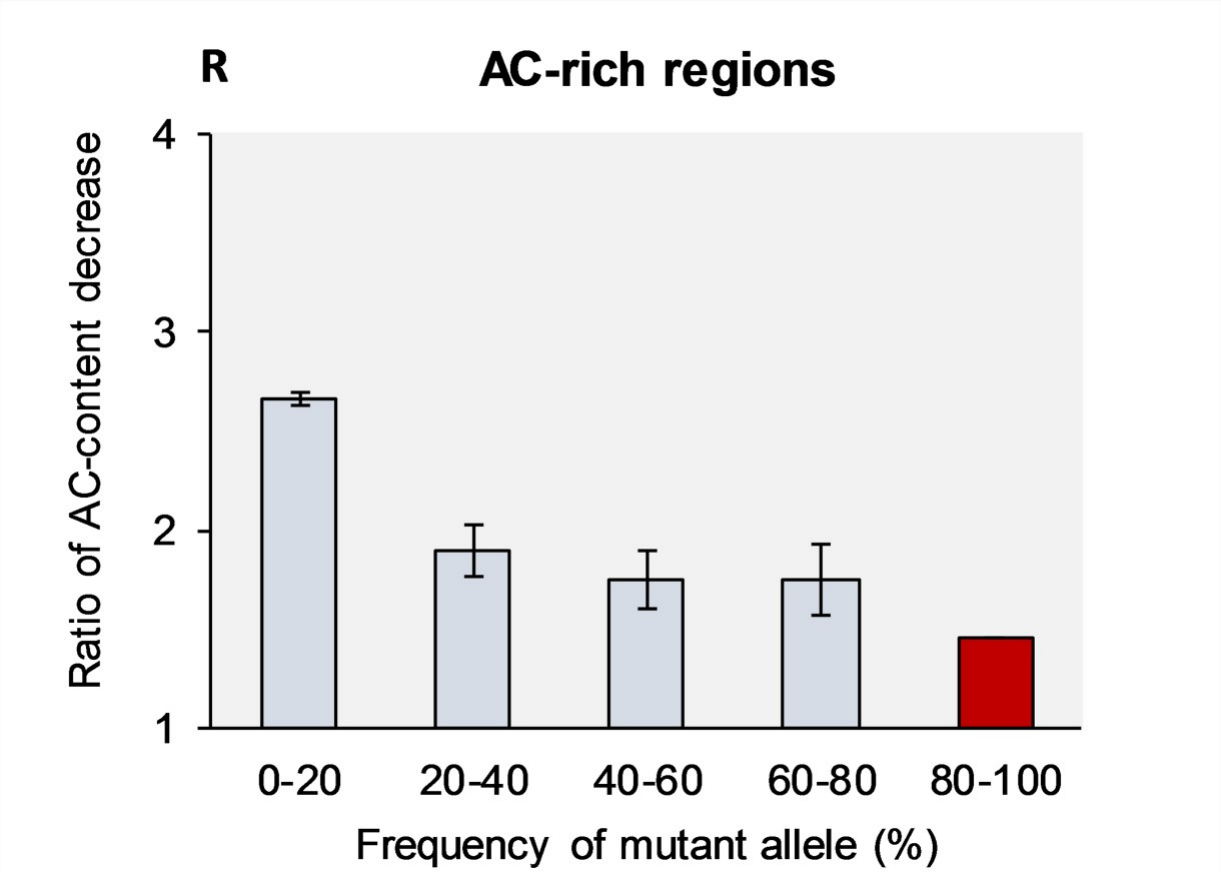
Ratio of CA-content decrease (R) in the AC-rich regions caused by mutations with various frequencies across populations. The R-ratio is the proportion between total number of mutations that change (A+C)->(T+G) vs. (T+G)->(A+C) mutations. The projected R-value for the fifth bin (80–100%) is shown in red, which does not have experimental support. Bars show standard error of the means, which is due to the limited sample size (see Methods section).

The mutation dynamics of novel and “old” alleles was studied for the least explored AC-rich regions–those chromosomal segments one DNA strand of which is predominantly composed by C and A nucleotides (the complementary strand is of cause TG-rich). These genomic regions have also distinctive DNA properties and their biological functions are only vaguely understood [13]. Table 5 shows that the frequency of various types of transitions and transversions inside AC-rich DNA strands may differ up to 2-3-fold from the average frequencies of these mutations over the entire genome (Table 1). For example, A→C is 2.3 times more frequent than A→T type of transversions in Table 5. This means that nucleotide context and DNA structure inside these regions critically influence mutation dynamics. Similar to R-rich regions, we observed that mutations on their path to fixation efforts to prevent the AC-rich DNA strands from degradation of their biased nucleotide compositions (about 30% difference between novel and nearly-fixed mutations). The ratio of AC-degradation is ***R***_(AC→GT)/(GT→AC)_ = 2.6 in the first bin and 1.6 in the fourth bin, see Fig 5.

## Discussion

### Extensive loss of G:C pairs in human genome via novel mutations

The normalized mutation ratio ***N***_(S→W)/(W→S)_ = 1.92 per site for the whole genome allows us to calculate the number of G:C pairs lost by brand new *de novo* mutations per individual. Let’s assume for simplicity that, on average, a human being has 100 *de novo* mutations [15]. About 16% of these mutations should be transversions (G→C; C→G; A→T; and T→A) that do not switch W and S base pairs, while the remaining eight types of substitutions, comprising 84 mutations, do switch between S and W base pairs (Table 1) [33]. Taking into consideration that ***N*** = 1.92 and global GC-content is 42% then, among these 84 *de novo* mutations, 49 change strong base pair into weak one (G:C→A:T), while 35 have the reverse effect: A:T→G:C. Hence, on average, the net effect of *de novo* mutations is that in each person 14 G:C pairs are converted into A:T pairs. Even if we take into consideration the lowest estimation for the amount of *de novo* mutations per individual (~50 mutations), then they still cause the noticeable overall conversion of seven G:C pairs into A:T pairs per individual. In both scenarios, the ratio ***N*** = 1.92 drives the genomic GC-percentage to 34.2% equilibrium (calculated using the proportions). However, Table 1 and Fig 1 testify that G:C→A:T mutations have disadvantages and/or A:T→G:C have advantages on their paths to fixation since the ratio (S->W)/(W->S) monotonously declines with increasing derived allele frequency. This effect completely stops the loss of G:C pairs at the stage of their fixation in the human genome. Therefore, at the current stage of evolution, there is a stable equilibrium of GC-content at 42%. Below we consider whether BGC theory is responsible for preservation of GC-content in humans.

### Effect of BGC on preservation of GC-content in humans

Let’s consider the average bias in BGC mismatch reparation (S→W)/(W→S) = 56:44, as evaluated by Dutta *et al*. This bias causes only one A:T base pair to be converted into G:C pair per gamete, according to the authors’ calculations [31]. This single W→S replacement is not enough to withstand the loss of 7–14 G:C pairs by novel mutations per individual. Even the highest estimated bias in BGC mismatch reparation of 68:32, calculated by Williams and others, would increase content by only 3 G:C base pairs per gamete, which is insufficient for preservation of genomic GC-content [30].

The impact of BGC on the increase of G:C content is proportional not only to the mis-match repair bias (W→S over S→W), but also to the total length of heteroduplexes formed during meiosis. In our calculations [31] we used the literature data for non-crossover heteroduplex average length of 75 nucleotides, which is considerably less than 1200 nucleotides for crossover heteroduplexes [34, 35]. Yet the non-crossover heteroduplexes twenty times outnumber the crossover ones, so their impacts are about the same [36, 37]. The problem here is that there are only a few experimental estimations of non-crossover heteroduplex lengths. These estimations are technically intricate, indirect, and imprecise. It is plausible to assume that non-crossover heteroduplex length in humans might be several times longer than 75 nucleotides. This conjecture would make BGC sufficient to convert 7–14 weak base-pairs into strong ones, which is the rate required to prevent loss of strong base-pairs by *de novo* mutations. Since weak to strong base pair replacements is only a fraction of all allele replacements caused by BGC, we conclude that, in total, BGC causes about 20–40 allele replacements per individual.

All in all, initially being skeptical to BGC capabilities, we acknowledge that reasonable re-evaluation of the biological parameters of this theory may make it strong enough to withstand bias in novel mutations and preserve the current equilibrium of GC-content in the entire human genome. It would also suggest length ranges for non-crossover heteroduplexes in humans.

In addition to recognized BGC evolutionary forces, there may be additional, still uncharacterized, molecular mechanisms that preserve genomic regions with biased nucleotide compositions (e.g. purine-rich or AC-rich) from evolutionarily mutational degradation.

### Natural selection

Natural selection is a well-established biological phenomoenon, that may be involved in preservation of particular nucleotides in the genome during evolution. Mathematical theories of population genetics, that try to describe natural selection, have been elaborated since the beginning of twentieth century. However, these theories include massive simplifications that consider discrete mutations one at a time, and do not address the dynamics of millions of mutations that co-exist simultaneously in each individual genome. For example, in a recent review McCandlish and Stoltzfus wrote: *“Even short-term models of population genetics often depend on radical simplifying assumptions such as neutrality*, *complete linkage (or full recombination)*, *the absence of epistasis (fitness interactions)*, *and so on”* [23]. Therefore, population genetics currently is unable to give us an accurate answer to how effective selection forces may be in withstanding deterioration of GC-composition by novel mutations in humans, when every individual has at least four million mutant alleles. Taking into account that each human individual has, on average, deleterious mutations in more than 20 genes [“*a typical genome contained 149–182 sites with protein truncating variants*” [22]], natural selection should be acting primarily on deleterious mutations that change proteins and, thus, reduce fitness [38, 39].

Computational modeling of genome evolution is another scientific tool that may help understanding the dynamics of numerous mutations. Currently, powerful supercomputers are capable to mimic the evolution of the entire genome dealing with millions of mutations. Our experiments with whole-genome computational modeling [40] showed evidence that an intense influx of *de novo* mutations causes the population fitness to decline. In other words, it is impossible to remove 10–20 unfavorable novel mutations per individual, even having optimal selection conditions. All in all, BGS with re-evaluated parameters looks to us to be the major plausible process responsible for the observed dramatic turnover of GC-composition.

### Maintenance of regions with extreme biased nucleotide compositions during evolution

Here we presented evidence suggesting that dynamics of SNP turnover may not prevent deterioration of extremely-biased nucleotide regions (GC-, AT-, R-, and AC-rich). Therefore, how have these regions been formed and maintained in mammalian genomes and those of other classes? The answer is very likely that all these genomic sequences with extremely-biased nucleotide compositions are composed up to 50% of simple repeats ([41] Table 3, p. 22). There are special evolutionary processes that occur within repetitive DNA sequences besides point mutations. Specifically, these are concerted evolution of tandem repeats and active insertion/deletion processes that elongate and shorten number of units of tandem DNA repeats. We assumed that the dynamics of simple repeats is responsible for maintenance of these genomic regions with biased nucleotide compositions [41].

## Materials and methods

The reference genome sequence GRch37 was downloaded from the UCSC genome browser from its ftp site (ftp://hgdownload.cse.ucsc.edu/goldenPath/hg19/chromosomes/) in fa.gz format and the allele frequency data from the 1000 Genomes Project phase 3 using the link ftp://ftp.1000genomes.ebi.ac.uk/vol1/ftp/release/20130502 in Variant Call Format (VCF) [22]. Ancestral allele information for the whole genome was obtained from the column 8 of the 1000 Genomes VCF file. These are represented as “AA =“ field in this column. Only high confidence ancestral alleles were considered which are represented by uppercase letters. Derived alleles were inferred based on the ancestral allele information. Only bi-allele SNPs with “PASS” flag were considered for further analysis. If the derived allele happens to be the alternative allele, then the frequency obtained from the “AF =“ field was considered as a derive allele frequency; else it was obtained by subtracting alternative allele frequency from 1. The number of derived alleles was calculated corresponding to the bins of range of frequencies using our new Perl program ***wg_analysis***. The ratios of GC-content decrease (***R-*** and ***N***-ratios) were calculated by the same program.

The human genome sequence was then computationally processed in order to characterize genomic-MRI regions (chromosomal sequences with uneven nucleotide compositions, which our lab studied for 15 years). We have generated a number of programs for studying these sequences and have good expertise on MRI sequences distribution and evolution [12, 14, 42]. For these reasons we specifically chose genomic-MRI regions for examination of nucleotide context effect on mutational dynamics. At first, the nucleotide composition was calculated for the 100-nucleotide sliding window, which is our default parameter for all types of genomic MRI fragments [42]. The thresholds for genomic-MRI regions were chosen based on our previous projects for consistency and were the following: 76% of G+C nucleotides in the window for CG-rich regions, 87% for A+T rich, 81% for CA rich; and 86% for the purine rich regions [12, 14]. These thresholds differ from each other because AT-composition is 1.38 times greater than GC-composition in the human genome and because various genomic-MRI regions have significantly different abundancies. These thresholds are consistent with our previous publications. The CA-rich and AG-rich regions were computed for single stranded DNA on the reference strand first and then on the complementary strand. When the segment of DNA inside the current window was characterized as genomic-MRI region, the window was extended in a cycle by 10 nucleotides until the overall composition began to fall below the assigned threshold. All the regions, their lengths, and nucleotide contents were calculated using our new Perl program ***regions*.*pl***.

The derived allele frequencies in genomic-MRI regions were calculated using a method similar to what was used for the whole genome. Then, the ratio of decrease of the nucleotide inhomogeneity (***R***-ratio) or ratio of GC-content decrease of these regions was calculated. This was performed using ***mri_analysis*.*pl*** program.

All computations were performed using Perl programs in a Linux workstation. The codes are available from our web page http://bpg.utoledo.edu/~afedorov/lab/prog.html.

The projected R- and N- values for the fifth bin (80–100% for mutant allele frequencies) were obtained using the trendline option in MS excel. The values were projected using linear (GC-rich, AT-rich) and logarithmic (AC-rich and purine rich) regression models. The best was chosen based on optimal R-square value.

The standard errors for the ‘AT → GC’ and ‘GC → AT’ events due to the limited numbers of observed cases (***N*** value, see below) were calculated with the formula using the Rule of Sample Proportions:

$$SE=\sqrt{\frac{p(1-p)}{N}}$$

where *N* is total number of gene conversion events (AT → GC and GC → AT), *p* is the proportion of AT → GC events and (1 –*p*) is the proportion of GC → AT events. These standard errors were used for calculation of uncertainty of R-Ratio values according to the formulas from [43] as described previously [12, 41]. Specifically, the propagation of uncertainty for a ratio *f* = *A/B* was calculated using the formula (σ*f*/*f*)2 = (σ*A*/*A*)2 + (σ*B*/*B*)2–2(σ*A*·σ*B*)/(*A·B*)·ρ*AB*, where ρ*AB* is the correlation coefficient for *A* and *B* variables which we assumed was negligible.

### Problems with ancestral/mutant alleles misidentification

The classification of SNP alleles as being ancestral or mutant is about 99% accurate. However, even 1% of errors in identification of ancestral/mutant status leads to a dramatic anomaly. Specifically, all SNPs with rare mutant alleles, for which ancestral/mutant status has been misidentified, are automatically interpreted as being “old” nearly-fixed SNPs and, thus, are allocated to the last bin (80–100% mutant allele frequency). This effect creates a strong distortion of data inside the last bin, due to the highest proportion of misidentified mutant/ancestral alleles being inside it. Because of this misidentification problem the dynamics of SNPs in the last bin contradicts several laws of population genetics. For example, the number of SNPs in this last bin should be lower than in any other bin. Instead, it is twice as large as the SNP number in the neighboring 60–80% bin (see Table 1). Due to these misidentification errors, predominantly concentrated inside 80–100% bin, we excluded the data for this last bin from all tables except rows 1–12 in Table 1. Instead, in our figures we projected the observed trends from the first four bins into the fifth one to get an idea what may be expected inside the last (80–100%) bin for mutations that are very close to fixation stage. Since extrapolated data for bin 5 do not have any experimental support, they must be treated with caution and, for this reason, they are marked by red color in the Figs 1–5.

## Supporting information

S1 Table (XLSX)

S2 Table Number of mutations inside human DNA repeats. Mutations are distributed among five bins based on the frequency of their mutant (derived) allele.
(XLSX)
